# Supplementary material for: Direct Photochemical Synthesis of Substituted Benzo[b]fluorenes
Source: Org Lett. 2024 Nov 25;26(48):10364–8. doi: 10.1021/acs.orglett.4c03978 (PMC11629382; doi:10.1021/acs.orglett.4c03978)
Supplement: Supplementary file 2 — ol4c03978_si_002.zip [file ol4c03978_si_002.zip › FID for Publication/5r/20240726_RC-2-C-5-133A_01/plots/RC-2-C-5-133A_PROTON_20240726_02_plot01.pdf]

Sample Name:
RC-2-C-5-133A
Data Collected on:
localhost.localdomain-vnmrs500
Archive directory:
/home/data/MBaumann/cscb500/RuairiCrawford/2024
Sample directory:
20240726_RC-2-C-5-133A_01
FidFile: RC-2-C-5-133A_PROTON_20240726_02

Pulse Sequence: PROTON (s2pul)
Solvent: acetone
Data collected on: Jul 26 2024

Temp. 25.0 C / 298.1 K
Sample #29, Operator: MBaumann

Relax. delay 1.000 sec
Pulse 45.0 degrees
Acq. time 1.822 sec
Width 4496.4 Hz
8 repetitions
OBSERVE H1, 499.7218396 MHz
DATA PROCESSING
FT size 16384
Total time 0 min 23 sec

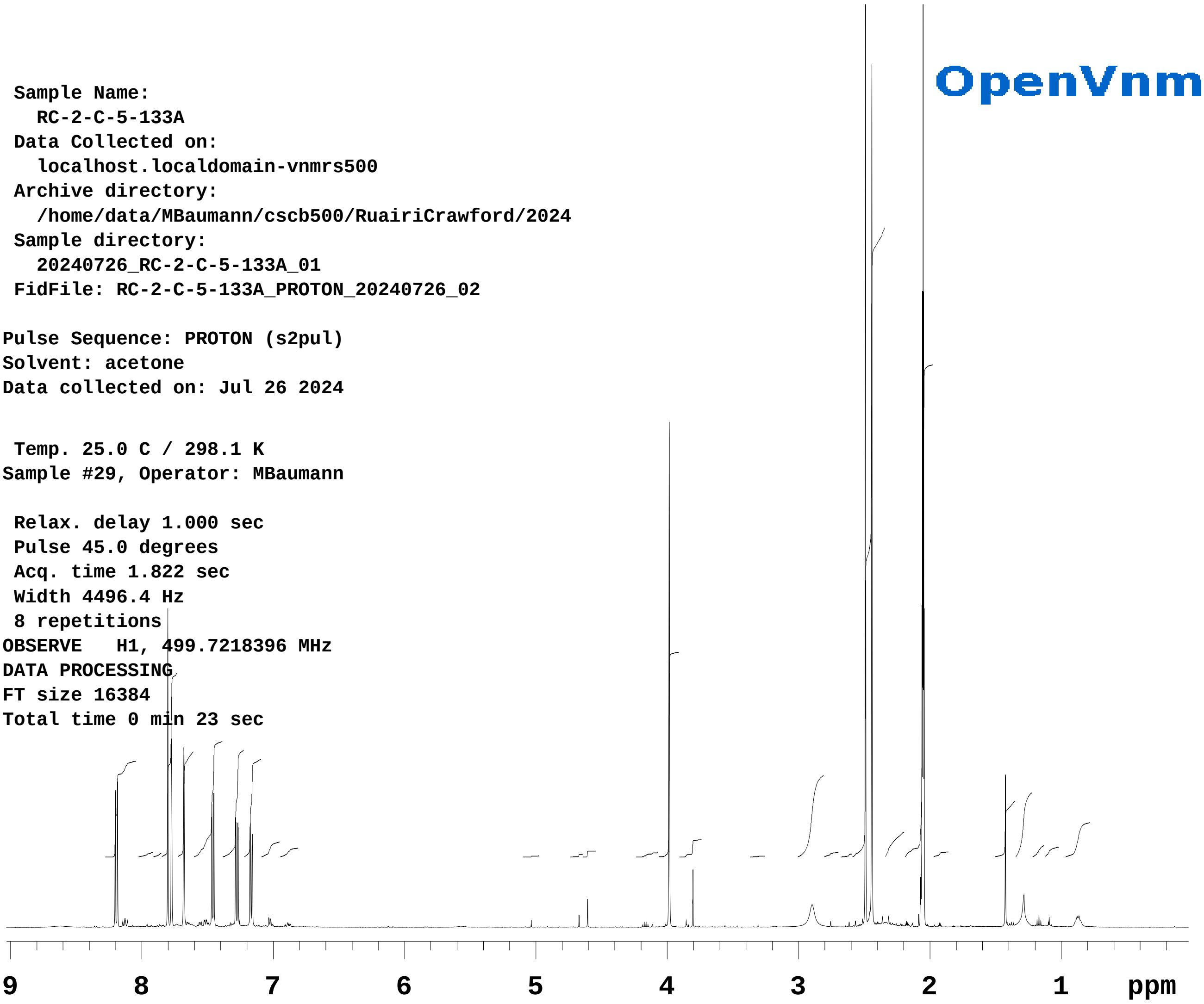

4.00 0.16 6.41 4.47 0.64 0.04 0.25 8.54 0.04 0.20 6.28 20.55 2.36 6.50 1.44

0.70 0.71 4.83 4.08 0.37 0.12 0.18 0.73 3.41 0.13 1.05 0.21 2.70 0.41

Plotname: RC-2-C-5-133A_PROTON_20240726_02_plot01
